# Do Weight Suppression and Body Mass Index Predict Daily Body Image and Eating Urges in Non‐Clinical Adults?

**DOI:** 10.1002/erv.70032

**Published:** 2025-09-25

**Authors:** An Dang, Matthew Fuller‐Tyszkiewicz, Litza Kiropoulos, Isabel Krug

**Affiliations:** ^1^ Melbourne School of Psychological Sciences The University of Melbourne Melbourne Victoria Australia; ^2^ School of Psychology Deakin University Geelong Victoria Australia; ^3^ Centre for Social and Early Emotional Development Deakin University Burwood Victoria Australia

**Keywords:** BMI, body dissatisfaction, eating disorders, weight suppression

## Abstract

**Introduction:**

The current study used ecological momentary assessment (EMA) to examine whether body mass index (BMI) and weight suppression (highest minus current weight) predicted momentary body dissatisfaction and disordered eating urges, including dietary restriction, excessive exercise, binge eating, and unhealthy eating, and whether trait eating disorder (ED) symptoms moderated these associations.

**Method:**

Data were collected from 686 adults (75% female), comprising community participants and undergraduate students, through six daily EMA surveys over seven days (42 possible assessments).

**Results:**

Multilevel models showed that lower BMI (*p* = 0.005) and greater weight suppression (*p* = 0.004) predicted higher average state body dissatisfaction, while higher BMI (*p* < 0.001) and greater weight suppression (*p* = 0.039) predicted stronger urges for unhealthy eating.

**Discussion:**

ED symptomatology moderated the relationship between BMI and dietary restraint, such that BMI positively predicted restraint urges at low levels of ED symptoms but negatively predicted them at high levels. No other moderating effects of ED symptomatology were observed for BMI or weight suppression on the remaining state‐based outcomes. Overall, both weight‐based severity indicators (BMI and weight suppression) demonstrated limited utility for indexing ED‐related state‐based variables in a female non‐clinical sample. Future studies should examine additional weight‐related severity indicators across both non‐clinical and clinical ED samples.

## Highlights

- Individuals with low BMI and higher weight suppression reported greater average body dissatisfaction across the week.
- Higher BMI and weight suppression were associated with increased urges for unhealthy eating, but not other disordered eating urges.
- Overall, BMI and weight suppression did not consistently predict state‐based body dissatisfaction or disordered eating urges, suggesting limited usefulness of these weight‐based indicators for understanding day‐to‐day eating disorder experiences.

## 1 Introduction

Eating disorders (EDs) are prevalent in both university and community populations, with up to 9% meeting criteria for a clinical diagnosis (Hay et al., 2023) and as many as 40% reporting subthreshold symptoms (Sparti et al. 2019). Both body mass index (BMI) and weight suppression (the difference between highest and lowest adult weight; Lowe 1993) have been found to be linked to body dissatisfaction and disordered eating urges (e.g., Duncan et al. 2017; Gorrell et al. 2019), two key risk factors for EDs (Stice et al. 2017). However, most studies on the predictive validity of BMI and weight suppression are cross‐sectional and focus on clinical ED samples (Dang et al. 2022, 2023). Given the variability and high prevalence of ED symptoms in community and university samples (e.g., Hay et al., 2023; Nagata et al. 2020), this study addresses gaps by examining how BMI and weight suppression predict changes in body dissatisfaction and disordered eating urges in a non‐clinical sample using an ecological momentary assessment (EMA) design.

### 1.1 Body Mass Index

Both the Diagnostic and Statistical Manual of Mental Disorders (DSM‐5; American and Psychiatric Association (APA) 2013; American Psychiatric Association 2022) and the International Classification of Diseases (ICD‐11; 2019) use BMI as a severity rating for anorexia nervosa (AN). The DSM‐5 categorises AN severity into four groups based on BMI: mild (≥ 17.0 kg/m^2^), moderate (16–16.99 kg/m^2^), severe (15–15.99 kg/m^2^), and extreme (< 15 kg/m^2^). The ICD‐11 classifies individuals with AN into ‘significantly low body weight’ and ‘dangerously low body weight’ using a cut‐off of BMI < 14.0 kg/m^2^. A recent meta‐analysis by Dang et al. (2022) examined severity indices across EDs. Within this review, five studies on AN (Dalle Grave et al. 2018; Gianini et al. 2017; Machado et al. 2017; Smith et al. 2017; Zayas et al. 2018) evaluated the DSM‐5 severity specifiers based on BMI and found limited support for their validity as indicators of ED psychopathology in AN. Notably, over 80% of the AN studies included in the Dang et al. (2022) review were based on clinical samples, limiting generalisability to community populations. Furthermore, while the DSM‐5 severity specifiers have received some empirical attention, there are currently no studies that have directly assessed the predictive validity of the ICD‐11 severity index for AN, despite its global importance as the primary classification system used by the World Health Organization.

Beyond the evaluation of diagnostic severity indicators, research has examined whether BMI alone predicts body dissatisfaction and disordered eating symptoms, with findings remaining inconsistent across both clinical and community settings. Whereas some studies (e.g., Duncan et al. 2017; Rø et al. 2012) reported a positive association between higher BMI and increased rates of disordered eating and ED risk, others (e.g., Argyrides et al. 2025; Stice 2002; Stice et al. 2017) found no significant link between BMI and body dissatisfaction or disordered eating. Taken together, these findings raise important questions about the utility of BMI as a standalone marker of ED severity across both clinical and community populations, underscoring the need for more nuanced, multidimensional approaches to assessing severity.

### 1.2 Weight Suppression

A key critique of using BMI to predict disordered eating and body dissatisfaction is its neglect of an individual's weight history (Dang et al. 2022). Weight suppression, linked to future weight gain and the development or worsening of ED symptoms, may be crucial in predicting the level of body dissatisfaction and disordered eating urges (Jennings et al. 2018; Gorrell et al. 2019). Being at a reduced body weight (i.e., weight suppressed) is thought to slow metabolism and create biological pressure to regain lost weight, increasing the risk of subsequent weight gain (Carter et al. 2008; Ochner et al. 2013), potentially leading to efforts to maintain weight suppression through restrictive eating and compensatory behaviours, which may contribute to ED symptoms (Juarascio et al. 2018).

Several studies support the biological and psychological significance of weight suppression in both clinical (e.g., Berner et al. 2013; Stice et al. 2020) and community‐based samples (e.g., Burnette and Mazzeo 2020). Gorrell et al. (2019) conducted a narrative review of 31 studies looking at the relationship between weight suppression and disordered eating and weight outcomes across both community and clinical‐based samples (e.g., AN, bulimia nervosa). They concluded that weight suppression is a useful index of ED symptom severity in clinical and community samples (Gorrell et al. 2019). Subsequent studies have further supported the role of weight suppression in indexing ED severity in both adults (Garber et al. 2019) and adolescents (Matthews et al. 2022) with clinical diagnosed EDs. Together, this evidence highlights weight suppression as a robust and transdiagnostic marker of ED severity, warranting its continued consideration in both research and clinical practice.

### 1.3 Ecological Momentary Assessment

Studies assessing the predictive validity of BMI (including DSM‐5 and ICD‐11 severity cut‐offs) and weight suppression have primarily relied on retrospective self‐report methods. While these provide insight into person‐level characteristics associated with ED symptomatology, they are prone to recall bias, with evidence showing low correspondence between trait and state measures of the same variables (e.g., Portingale et al. 2022). To overcome these limitations, research has increasingly employed EMA to capture real‐time fluctuations in disordered eating and body dissatisfaction while minimising recall bias (Shiffman et al. 2008; Lantz et al. 2018; Portingale et al. 2022).

To date, only one EMA study (Fuller‐Tyszkiewicz et al. 2020) has examined links between state‐based variables (e.g., mood, body dissatisfaction, appearance comparisons) and trait‐based ED severity in a community sample of 260 women, assessed with the Eating Attitude Test‐26 (EAT‐26; Garner et al. 1982). State‐based variables explained 34% of variance in EAT‐26 scores. However, this study was limited to an all‐female community sample and did not include BMI or weight suppression, which are key physiological indicators of severity in restrictive EDs (e.g., AN). Despite their relevance, no study has tested whether BMI and weight suppression predict momentary, state‐based ED symptoms in a non‐clinical sample (Dang et al. 2022; Dang et al. 2022).

### 1.4 Limitations and Gaps in the Literature

The current literature is limited in several important ways. First, previous studies have focused predominantly on clinical ED samples, leaving little understanding of how BMI and weight suppression operate in non‐clinical populations. Examining severity in non‐clinical samples is critical for identifying early risk markers and informing prevention efforts before symptoms escalate to clinical levels.

Second, studies assessing the predictive validity of BMI for body dissatisfaction and disordered eating have frequently relied on the DSM‐5's arbitrary BMI cut‐off values, which lack empirical grounding (Dang et al. 2022). Future studies should therefore test BMI as a continuous variable rather than relying on categorical thresholds, which risk obscuring meaningful variation in severity. Overall, these gaps highlight the need to investigate whether BMI and weight suppression, when assessed as continuous variables, can meaningfully predict state‐based outcomes such as body dissatisfaction and disordered eating urges using EMA in non‐clinical samples.

### 1.5 The Current Study

This study used an EMA design to examine whether BMI and weight suppression, assessed as continuous variables, predicted daily body dissatisfaction and disordered eating urges in a non‐clinical sample. Disordered eating urges were used as proxies for behavioural engagement and analysed across dietary restraint, excessive exercise, binge eating given their distinct functions in managing body‐related distress (Fitzsimmons‐Craft et al. 2016; Gonçalves and Gomes 2012; Tasca et al. 2009). Furthermore, urges to overeat were assessed as a proxy for binge eating behaviours, as they represent a key motivational aspect of binge eating (Goldschmidt et al. 2014).

Another aim was to examine whether baseline ED symptoms assessed through the EAT‐26, moderated the effects of BMI and weight suppression on daily body dissatisfaction and disordered eating urges. This builds on prior research (e.g., Coffino et al. 2016) suggesting that individuals with higher ED symptoms may be more sensitive to these weight‐related factors.

Overall, based on the current literature, it was hypothesised that: (H1) lower BMI and higher weight suppression would predict greater daily body dissatisfaction and disordered eating urges, and (H2) these associations would be stronger among individuals with higher baseline ED symptoms. This study addresses gaps in prior cross‐sectional and clinical ED research by testing associations between BMI and weight suppression in real‐world, non‐clinical settings using EMA, with implications for clinical assessment and early prevention and intervention.

## 2 Method

### 2.1 Participants

Participants were recruited after Human Research Ethics Committee approval, through the University's Research Experience Program for students and via social media platforms to reach a wider community sample. Out of 1932 baseline survey respondents, 969 completed the EMA phase. To avoid bias from systematic completion differences, 283 participants who completed less than 50% of EMA surveys were excluded (Shiffman et al. 2008). The remaining participants provided a comparable number of surveys to those in similar EMA studies (e.g., Fuller‐Tyszkiewicz et al. 2018).

The final sample comprised 686 participants (mean age = 19.95, SD = 4.54; mean BMI = 22.14 kg/m^2^, SD = 4.33) aged 18 to 76, with 37 self‐reporting a lifetime ED diagnosis. Most participants were female (75%), single (69%), had completed year 12 or less (83%), and identified as Asian (55%). Detailed demographic characteristics are outlined in Table 1. Using the powerlmm package in R to estimate post‐hoc power for a MLM, the final sample of 686 participants was sufficient to detect small effects (> 5% variance explained) with > 0.80 power (*α* = 0.05).”

**TABLE 1:** Demographic characteristics of the total sample.

Demographic variables	Statistics
Age (M ± SD)	19.95 ± 4.54
Gender (%)	
Female	516 (75%)
Male	161 (23%)
Others	9 (1%)
Sexual orientation (%)	
Heterosexual	523 (78%)
Homosexual	28 (4%)
Bisexual	96 (14%)
Asexual	10 (1%)
Others	16 (2%)
Ethnicity (%)	
Caucasian	242 (35%)
Southern Asian/Southeast Asian	200 (29%)
Easter Asian	178 (26%)
Others	66 (10%)
Marital status (%)	
Single	474 (69%)
In a relationship	198 (29%)
Married	12 (1.7%)
Others	2 (0.3%)
Education (%)	
Year 12 or below	605 (83%)
Certificate	10 (1.3%)
Diploma	23 (3.3%)
Bachelor's degree	71 (9.7)
Postgraduate	20 (2.7%)
Eating disorder diagnoses (%)	
No	649 (95%)
Yes	37 (5%)

Abbreviation: ED, Eating Disorder.

### 2.2 Procedure

#### 2.2.1 Phase 1: Trait‐Based Assessment

After signing up, participants received a web link to an online survey containing study information and a consent form. Upon providing consent, they entered their email and a self‐generated ID to link Phase 1 (trait) and Phase 2 (EMA) data. They then completed a baseline survey (see 2.3.1.) and were then emailed instructions for downloading and using the custom‐built EMA app, SEMA3.

#### 2.2.2 Phase 2: Ecological Momentary Assessment

**FIGURE 1:**
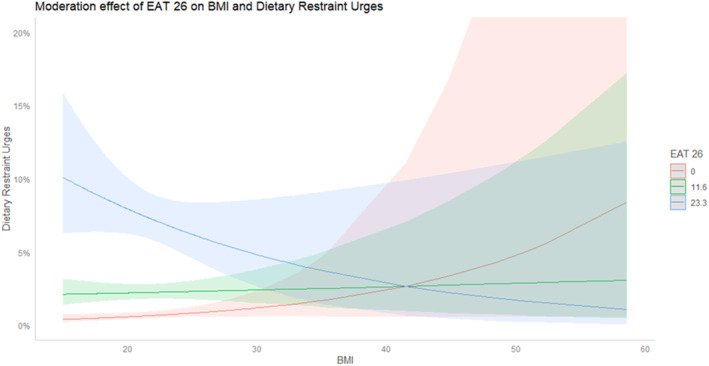
Moderation effect of EAT‐25 on the relationship between BMI and dietary restraint urges.

The morning after completing Phase 1, SEMA3 sent push notifications six times per day at semi‐random intervals (every 1–2 h) between 9:00 a.m. and 10:00 p.m. for seven days (up to 42 assessments). Participants completed a brief 1–2‐min survey on body dissatisfaction and disordered eating urges. Random intervals aimed to reduce response bias, while the brief survey minimized participant burden (Fuller‐Tyszkiewicz et al. 2020). Participants had a 30‐min window to complete each survey before it expired and was marked as missing. Researchers provided support and sent up to two reminders over 7 days to those with less than 50% compliance (Chia et al. 2018). Participants were debriefed upon study completion. Students received up to two hours of course credit based on their EMA compliance and participants from the community had the option to enter a draw to win one of five AUD100$ e‐gift cards (see flowchart, Supporting Information S1: Figure 1).

### 2.3 Measures

#### 2.3.1 Baseline Measures

##### 2.3.1.1 Demographics

Participants provided self‐reported information on various demographic‐related variables, including their age, gender, sexual orientation, current height, highest weight, current weight, and lowest weight (used to calculate BMI, and weight suppression), primary language, cultural background, marital status, the highest level of education completed, and whether they had a self‐reported lifetime diagnosis of an ED (current or prior).

##### 2.3.1.2 Eating Pathology

Participants' attitudes and behaviours related to eating were evaluated using the EAT‐26 (Garner et al. 1982). The EAT‐26 comprises 26 items rated on a 6‐point scale, ranging from 0 = never to 5 = always, that measures disordered eating symptoms. In the present study, the internal consistency of the total EAT‐26 score was strong (Cronbach's alpha = 0.93).

#### 2.3.2 Ecological Momentary Assessment Measures

##### 2.3.2.1 Body Dissatisfaction

Participants were asked about their current satisfaction with their appearance (i.e., *‘How satisfied are you with your appearance right now?*’) using an 11‐point scale ranging from 0 (completely dissatisfied) to 10 (completely satisfied). The responses were reverse scored so that a higher score indicated a higher level of body dissatisfaction. This single‐item measurement was adapted from previous EMA studies in the ED literature that examined body dissatisfaction (Fuller‐Tyszkiewicz et al. 2020; Portingale et al. 2022; Liu et al. 2022; Martin et al. 2023).

##### 2.3.2.2 Urges to Engage in Disordered Eating

Participants were asked to report whether they had experienced urges to engage in specific disordered eating behaviours since the previous survey. These behaviours included: (a) ‘*Urge to consciously restrict food intake to control weight/shape*’ (dietary restraint), (b) *‘Urge to engage in at least 15 min of exercise to control weight/shape*’ (exercise), (c) ‘*Urge to eat a large amount of food relative to what others would eat in the same situation/time*’ (binge eating), and (d) ‘*Urge to eat unhealthy food*’. Participants responded with a code of 1 for ‘yes’ and 0 for ‘no’. Similar items have been utilised in prior studies to evaluate disordered eating urges and have shown validity in measuring such constructs (Fitzsimmons‐Craft et al. 2016; Martin et al. 2023; Portingale et al. 2022).

### 2.4 Statistical Analysis

#### 2.4.1 Preliminary Analyses

Prior to the main analyses, the quality of the baseline and EMA data was evaluated. There was no missing data among BMI, weight suppression, trait ED psychopathology (measured by the EAT‐26) as participants were required to answer all questions during Phase 1 via the Qualtrics platform.

There were also no missing data in Phase 2 of the EMA, but participants varied in the number of completed surveys out of 42. Preliminary tests checked for biases by analysing compliance rates using Pearson's correlation or ANOVA with baseline demographic and predictor variables to find differences between those who completed over 50% and those with less than 50% compliance.

Second, all EMA‐assessed variables used as outcomes in the hypothesis testing (e.g., state body dissatisfaction) were examined for time‐related and reactivity effects. This involved inspecting whether reports of these outcomes differed based on the time of day (in hourly blocks), day of the week (weekday vs. weekend), and/or the order of assessment throughout the EMA phase. Any significant time‐related or reactivity effects were included as covariates in the final models to account for their effects on the modelled outcome variables.

#### 2.4.2 Main Analyses

Multilevel modelling was employed for hypothesis testing to account for the non‐independence of data resulting from repeated assessments during the EMA phase. We employed a bottom‐up approach to data analysis, gradually increasing the complexity of our models. First, null models ‐ only including Level 1 outcome variables (state body dissatisfaction and disordered eating urges)—were tested to explore between‐person differences in average levels for these constructs relative to within‐person fluctuations. To assess within‐person variability, we applied group mean centring to the Level 1 outcome variables, specifically body dissatisfaction and disordered eating urges. Multilevel modelling was only utilised when significant variance was observed across individuals, based on intraclass correlation coefficients (ICC) exceeding 5%.

Next, the Level 2 predictors (BMI and weight suppression) were individually included in the models to evaluate their effects on predicting the average of Level 1 outcome variable (rather than state‐level changes in these outcomes). Finally, we assessed the moderating effect of the Level 2 trait EAT‐26 total score on the relationships between both BMI and weight suppression and the tested Level 1 outcome variables (body dissatisfaction and disordered eating urges). Models employed specific distributions based on the type of outcome. Binomial models were assumed for categorical data (e.g., disordered eating urges), while Gaussian models were assumed to continuous data (e.g., body dissatisfaction). All data pre‐processing and analyses were conducted using RStudio version 4.0.2. *p* value of < 0.05 was considered statistically significant.

To allow direct comparison of BMI and weight suppression in predicting state‐based variables, both were standardised into z‐scores (with a mean of 0 and SD of 1), enabling their beta coefficients to be compared on the same scale.

## 3 Results

### 3.1 Preliminary Analyses

#### 3.1.1 Compliance

On average, participants completed 33.21 (SD = 6.15) out of the 42 possible EMA surveys, indicating a compliance rate of 79.07%. There were no significant associations between compliance rates for EMA surveys and BMI (*p* = 0.546), weight suppression (*p* = 0.091), EAT‐26 total score (*p* = 0.277), or various demographic‐related variables, including age (*p* = 0.254), ethnicity (*p* = 0.414), education (*p* = 0.517), gender (*p* = 0.865), sexual orientation (*p* = 0.764), marital status (*p* = 0.051) and self‐reported ED lifetime diagnosis (*p* = 0.734).

#### 3.1.2 Time‐Related and Reactivity Effect

Significant reactivity effects were found for all Level 1 outcome variables (see Table 2). Specifically, throughout Phase 2, scores for body dissatisfaction (*p* < 0.001) increased, while urges for dietary restraint (*p* < 0.001), excessive exercise (*p* < 0.001), and unhealthy eating (*p* < 0.001) decreased. It was also observed that lower urges for dietary restraint (*p* < 0.001), excessive exercise (*p* < 0.001), unhealthy eating (*p* < 0.001), and higher urges for binge eating (*p* = 0.026) were reported later in the day. A significantly higher body dissatisfaction was reported on weekdays compared to weekends (*p* = 0.007). Therefore, to account for these effects, the order of assessment, time of day, and day of week were included as covariates in the main analyses.

**TABLE 2:** Effects of order of assessment, time of day, and day of week on outcomes.

Predictors	Body dissatisfaction			Negative mood			Urges for exercise			Urge for dietary restraint			Urges for binge eating			Urges for healthy eating		
	*b*	*T*	*P*	*b* (95% CIs)	*t*	*p*	*b*	*t*	*p*	*b*	*t*	*p*	*b*	*t*	*p*	*b*	*t*	*p*
Order of assessment	0.009	7.97	< 0.001	0.009	8.08	< 0.001	−0.001	−7.83	< 0.001	−0.001	−6.17	< 0.001	−0.00008	−0.0723	0.0469	−0.001	−6.38	< 0.001
Time of day	−0.006	−1.96	0.050	−0.020	−6.44	< 0.001	−0.003	−6.81	< 0.001	−0.002	−5.279	< 0.001	0.001	2.39	0.016	−0.002	3.95	< 0.001
Day of week	0.066	2.676	0.007	−0.044	−1.73	0.083	0.004	1.16	0.245	0.001	0.264	0.792	−0.001	−0.21	0.834	−0.007	−1.53	0.127

*Note: b* = unstandardised coefficients, *t* = t‐values for continuous variable.

### 3.2 Main Analyses

#### 3.2.1 Descriptive Statistics

Descriptive statistics of both Level 1 and Level 2 variables (Table 3), including means, SDs, and the possible range of continuous variables, as well as frequencies for categorical variables, are presented in Table 3. There was no concern with multicollinearity among the predictors as the correlations between these variables were small (see Supporting Information S1: Figure 2). On average, moderate levels of body dissatisfaction were observed in the EMA assessments. Urges for binge eating were reported relatively infrequently (7%), while urges for dietary restraint (35%) and exercise (28%) were more frequently reported. Additionally, 19% of the participants were found to have exhibited clinically significant levels of trait ED symptoms based on their scores on the EAT‐26 (≥ 20).

**TABLE 3:** Descriptive statistics for Level 1 and Level 2 variables.

Variables	M ± SD	ICC_BP_	Range
Level 1			
Body dissatisfaction	5.09 ± 2.34	0.51	0–10
Urges for dietary restraint (*n*, %)	177 (35%)	0.39	0–1
Urges for exercise (*n*, %)	190 (28%)	0.29	0–1
Urges for healthy eating (*n*, %)	182 (27%)	0.24	0–1
Urges for binge eating (*n*, %)	49 (7%)	0.21	0–1
Level 2			Min‐Max
BMI	22.14 ± 4.33	—	15.1–58.6
Weight suppression	4.96 ± 5.08	—	0–66
EAT‐26	11.56 ± 11.63	—	0–65

*Note:* EAT‐26, Eating Attitudes Test‐26% represents the proportion of ‘yes’ responses.

#### 3.2.2 Multilevel Modelling

##### 3.2.2.1 (H1) BMI and Weight Suppression Predicting Daily Changes in Body Dissatisfaction, and Disordered Eating Urges

A lower BMI predicted greater average levels of daily body dissatisfaction (*p* = 0.005), and lower urges of having unhealthy eating (*p* < 0.001; Table 4).

**TABLE 4:** Fixed effects for BMI and weight suppression, predicting outcomes and moderation effects of EAT‐26 on the tested relationships.

Predictors	Body dissatisfaction				Urge for dietary restraint				Urges for excessive exercise				Urge for binge eating				Urges for unhealthy eating			
	*b*	*Se*	*p*	*R* ^2^	*b*	*se*	*p*	*R* ^2^	*b*	*se*	*p*	*R* ^2^	*b*	*se*	*p*	*R* ^2^	*b*	*Se*	*p*	*R* ^2^
BMI	−0.13	0.049	**0.005**	0.070	0.17	0.12	0.134	0.008	0.15	0.09	0.102	0.014	0.17	0.24	0.129	0.004	0.26	0.08	**<** **0.001**	0.017
Weight suppression	−0.14	−0.005	**0.004**	0.060	0.08	0.12	0.498	0.006	0.13	0.09	0.158	0.013	0.21	0.11	0.067	0.006	0.16	0.08	**0.039**	0.010
BMI × EAT‐26 total	−0.001	−0.004	0.797	0.217	−0.02	0.01	**0.021**	0.225	−0.02	0.01	0.078	0.020	−0.002	0.01	0.866	0.091	0.01	0.01	0.516	0.060
Weigh suppression × EAT 26 total	−0.004	−0.004	0.342	0.151	−0.01	0.01	0.182	0.218	0.001	0.01	0.890	0.105	−0.004	0.01	0.670	0.093	0.01	0.01	0.282	0.045

*Note:* *p* < 0.05 is in bold.

Abbreviation: EAT‐26, Eating Attitudes Test‐26.

A lower level of weight suppression was found to be associated with higher average levels of daily body dissatisfaction (*p* = 0.004) and lower urges to engage in unhealthy eating (*p* = 0.039) throughout the 1‐week EMA period. No other significant associations were identified between BMI and weight suppression with the other state‐based EMA variables.

##### 3.2.2.2 (H2) Moderating Effects of Trait Eating Disorder Symptoms

Moderation analyses revealed a significant moderation effect of the overall EAT‐26 score on the relationship between BMI and urges for dietary restraint (*p* = 0.021). Specifically, BMI was found to have a positive relationship with dietary restraint at low levels of disordered eating symptomatology and a negative relationship with dietary restraint at high levels of disordered eating symptomatology (see Figure 1).

## 4 Discussion

The current EMA study explored for the first time whether BMI and weight suppression predicted average daily body dissatisfaction and disordered eating urges over 7 days in a predominantly female university sample. Findings offered limited support for these predictors: lower BMI and weight suppression were linked to higher daily body dissatisfaction, and both were associated with greater daily urges for unhealthy eating. Our second hypothesis was partially supported, with ED symptomatology moderating the relationship between BMI and dietary restraint, though no other moderating effects of ED symptomatology were observed for BMI or weight suppression on the remaining state‐based outcomes.

### 4.1 Predictive Validity of BMI on State Body Dissatisfaction and Disordered Eating Urges

Consistent with previous studies (e.g., Porras‐Garcia et al. 2020), we found that those with a lower BMI tended to report greater daily body dissatisfaction relative to those with a higher BMI. Although the exact mechanisms behind this relationship remain unclear, previous studies have suggested that individuals with a lower BMI tend to overestimate their body size and shape, while those with a higher BMI might provide an underestimation (Cornelissen et al. 2015; Porras‐Garcia et al. 2020).

However, there are also studies (e.g., Jones et al. 2019; Kogure et al. 2019) that found contradicting results to ours where individuals with a higher BMI were more likely to experience higher levels of body dissatisfaction. Such disparities can be attributed to differences in the methodology or the sample characteristics (e.g., university students vs. general or clinical ED populations). Specifically, our study primarily involved university students with a mean BMI in the normal range (22.14 kg/m^2^), unlike prior research with samples in the overweight range (e.g., 29.1 kg/m^2^; Kogure et al. 2019), so our finding that BMI predicts body dissatisfaction may only generalise to similar populations.

Another important finding was that a lower BMI was related to greater urges to engage in unhealthy eating. This finding contradicts the existing literature, which has shown a significant positive association between BMI and unhealthy eating behaviours (Guertin and Pelletier 2021; Heerman et al. 2017). It is important to note, however, that previous studies have primarily focused on individuals' actual engagement in unhealthy eating behaviours, whereas our study assessed only the presence of urges to engage in such disordered eating behaviours. The relationship between urges and actual behaviours can be influenced by factors such as preoccupation with food and eating as well as dietary restraint. Nevertheless, the complexity of such relationships should be further investigated in future research.

### 4.2 Weight Suppression Predicting Body Dissatisfaction and Disordered Eating Urges

Inconsistent with our predictions and the literature (e.g., Berner et al. 2013; Burnette et al. 2018), lower weight suppression was found to be associated with a higher average daily level of body dissatisfaction. Such discrepancy from previous studies can be once again attributed to the lower mean weight suppression observed in our predominant university student sample (*M* = 4.96) compared to similar community‐based studies, where mean weight suppression ranged were from 8.19 to 12.85 (e.g., Chen et al. 2022; Burnette et al. 2018).

Differences in mean weight suppression between our study and prior research may be due to our predominantly female (75%) and Asian (55%) participant makeup, compared to earlier studies with fewer Asian participants (e.g., 7.8% in Burnette et al.) and more males (e.g., 48% in Chen et al. 2022). Our findings are therefore most applicable to individuals with similar characteristics, highlighting the need for future research to determine whether a specific threshold of weight suppression may reverse expected effects, such as lower weight suppression predicting greater body dissatisfaction.

Weight suppression was only found to significantly predict higher daily urges to eat unhealthy food, with a small effect size (*β* = 0.16). This finding aligns with the concept that those who suppress their weight, often through dieting and inappropriate compensatory actions, are more inclined to fixate on food, especially unhealthy choices (Butryn et al. 2011). However, weight suppression did not associate with any of the remaining tested disordered eating urges, thus offering limited evidence for its validity in predicting disordered eating urges within natural environments.

While previous research has supported weight suppression as a predictor of body dissatisfaction and disordered eating urges (Gorrell et al. 2019), it did not outperform BMI in this study. This may be due to the use of continuous BMI values, which offered greater statistical sensitivity than categorical cut‐offs used in past research (e.g., Dang et al. 2022). Additionally, the study did not account for the duration of weight loss, which may influence the psychological impact of weight suppression. For instance, individuals who lost 4 kg over 4 weeks are likely to face higher disordered eating urges, body dissatisfaction, and psychological and medical risks compared to those who lost the same amount over 6 months (Jaime and Mank 2024; Garber et al. 2019; Whitelaw et al. 2018). Future studies should consider weight loss duration and identify empirically based severity cut‐offs to improve the predictive utility of these indicators.

### 4.3 The Moderating Effect of Trait Eating Disorders Symptoms on the Relationship Between BMI and Disordered Eating Urges

Our second hypothesis was partially supported, in that ED symptomatology—measured by the EAT‐26—moderated the BMI‐dietary restraint relationship. While in the current study, BMI alone was not a strong predictor of urges to engage in dietary restraint, there was a significant BMI‐ED symptomatology interaction. BMI positively influenced dietary restraint urges at low ED symptomatology levels (i.e., low EAT‐26 trait score) but negatively affected it at high ED symptomatology levels (i.e., high EAT‐26 trait score). However, we did not identify any other moderating effects of EAT‐26 on the tested relationships between our predictors (BMI and weight suppression) and our state‐based outcome variables (e.g., body dissatisfaction and other disordered eating urges). Future studies should examine other potential moderators, such as overvaluation of weight and shape or drive for thinness, which may influence the links between BMI, weight suppression, and state‐based body dissatisfaction and disordered eating urges (e.g., Chernyak and Lowe 2010; Dang et al. 2022) but have so far been unexplored.

### 4.4 Implications

Although the current findings require replication, several research and clinical implications emerge. Our results provide limited support for BMI and weight suppression as reliable indicators of state‐based body dissatisfaction and ED severity in a mixed student‐community sample. No single physiological marker can currently inform clinicians about the level of body dissatisfaction or disordered eating urges in non‐clinical populations, underscoring the need for comprehensive community screening. Such assessments should integrate biological factors (e.g., potassium, magnesium; Quesnel et al. 2023) and cognitive factors (e.g., drive for thinness, overvaluation of weight and shape; Krug et al. 2021) that may signal the need for early intervention. This is particularly important, as delays in treatment contribute to a more protracted illness course (Austin et al. 2021). Ideally, a combination of self‐report questionnaires, clinical interviews, and physiological measures should be employed to inform tailored prevention and early intervention strategies.

### 4.5 Strengths, Limitations and Future Direction

The relatively large non‐clinical sample strengthens the reliability of findings; however, several limitations should be noted. First, EMA items were assessed using single‐item measures to reduce participant burden, which may limit construct precision despite prior use (Portingale et al. 2022). In particular, the EMA binge eating item did not capture loss of control—an essential diagnostic criterion for clinical binge eating (American Psychiatric Association 2022)—which limits comparability with clinical definitions. However, this approach was appropriate given the focus on a non‐clinical sample. Future studies should incorporate EMA items that assess both overeating and loss of control, adopt validated EMA‐adapted scales (e.g., Body‐Image Ideals Questionnaire; Cash 2000), and pre‐register hypotheses to improve transparency.

Second, only disordered eating urges—not behaviours—were measured, which may not capture actual engagement. Future work should examine behavioural frequency and consider cognitive moderators such as drive for thinness (Krug et al. 2021) and shape/weight concerns (Dang et al. 2022; Gianini et al. 2017), as well as correlates of body dissatisfaction including self‐esteem (Cruz‐Sáez et al. 2020), social comparison (Ryding and Kuss 2020), and negative affect (McLean et al. 2010).

Third, BMI and weight suppression were based on self‐report, potentially reducing accuracy; weight suppression levels were also lower than in prior studies (Chen et al. 2022), highlighting the need for objective assessment. Finally, given the young, predominantly female student sample (*M* = 19.95, SD = 4.54), developmental weight suppression (Singh et al. 2021) may provide a more sensitive index, and future research should incorporate age at highest weight and recruit more diverse populations (Murray et al. 2017).

## 5 Conclusion

This study advances the ED severity literature by applying an EMA design within a non‐clinical sample. While BMI and weight suppression showed limited utility in predicting state‐based body dissatisfaction and disordered eating urges, our findings underscore the limitations of relying on weight‐based indicators alone. To refine our understanding of ED severity, future research must adopt longitudinal EMA approaches and recruit more diverse populations (e.g., males, different age groups, individuals from varied cultural and ethnic backgrounds). Clinicians are urged to move beyond weight‐focused markers and instead employ comprehensive, multidimensional assessments to better identify risk for body dissatisfaction and disordered eating, and to guide timely prevention and interventions.

## Supporting information


Supporting Information S1
