# Supplementary material for: Do Weight Suppression and Body Mass Index Predict Daily Body Image and Eating Urges in Non‐Clinical Adults?
Source: Eur Eat Disord Rev. 2025 Sep 25;34(2):347–57. doi: 10.1002/erv.70032 (PMC12862546; doi:10.1002/erv.70032)

**Supplementary Figure 1**

Data collection stages


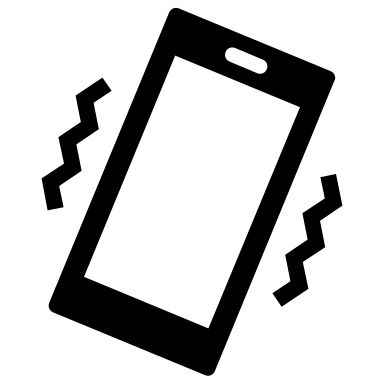


After 7 days, participants were debriefed and reimbursed.

Research assistant send reminders to complete survey (no more than one per 2 days)

For participants who have lower than 50% compliance rate

**Phone**: push notifications x 6 (randomly from 9am to 10pm) to complete 1-2 min survey (Maximum number of surveys = 42)

**Phase 1**

Qualtrics survey to measure trait variables (BMI, weight suppression and EAT-26)

**Phase 2 (EMA phase using SEMA app)**

Using single-item to measure body dissatisfaction, disordered eating urges

)

)

**Supplementary Figure 2**

Correlation matrix between predictors and outcome variables


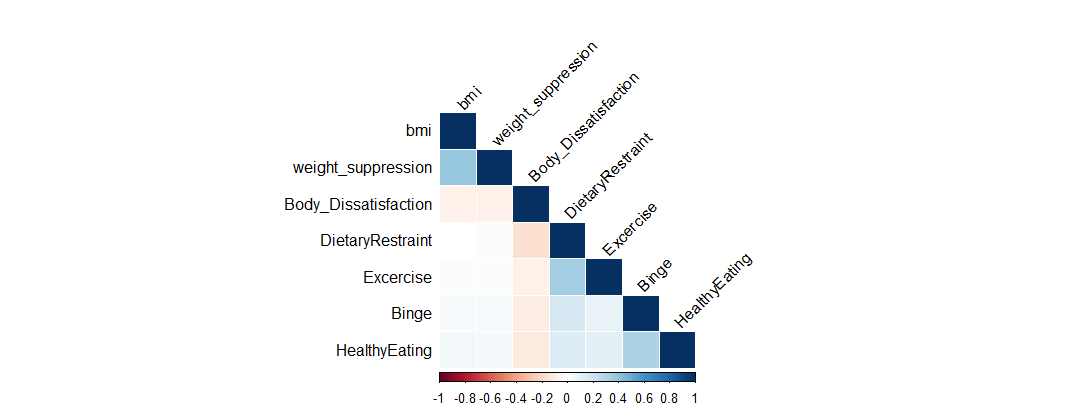

Supplement: Supplementary file 1 — Supporting Information S1 [file ERV-34-347-s001.docx]
